# Learning to Use Illumination Gradients as an Unambiguous Cue to Three Dimensional Shape

**DOI:** 10.1371/journal.pone.0035950

**Published:** 2012-04-30

**Authors:** Glen Harding, Julie M. Harris, Marina Bloj

**Affiliations:** 1 Bradford School of Optometry and Vision Science, University of Bradford, Bradford, United Kingdom; 2 Vision Lab, School of Psychology, University of St. Andrews, St Andrews, United Kingdom; Université Paris 5, and CNRS, France

## Abstract

The luminance and colour gradients across an image are the result of complex interactions between object shape, material and illumination. Using such variations to infer object shape or surface colour is therefore a difficult problem for the visual system. We know that changes to the shape of an object can affect its perceived colour, and that shading gradients confer a sense of shape. Here we investigate if the visual system is able to effectively utilise these gradients as a cue to shape perception, even when additional cues are not available. We tested shape perception of a folded card object that contained illumination gradients in the form of shading and more subtle effects such as inter-reflections. Our results suggest that observers are able to use the gradients to make consistent shape judgements. In order to do this, observers must be given the opportunity to learn suitable assumptions about the lighting and scene. Using a variety of different training conditions, we demonstrate that learning can occur quickly and requires only coarse information. We also establish that learning does not deliver a trivial mapping between gradient and shape; rather learning leads to the acquisition of assumptions about lighting and scene parameters that subsequently allow for gradients to be used as a shape cue. The perceived shape is shown to be consistent for convex and concave versions of the object that exhibit very different shading, and also similar to that delivered by outline, a largely unrelated cue to shape. Overall our results indicate that, although gradients are less reliable than some other cues, the relationship between gradients and shape can be quickly assessed and the gradients therefore used effectively as a visual shape cue.

## Introduction

**Figure 1:**
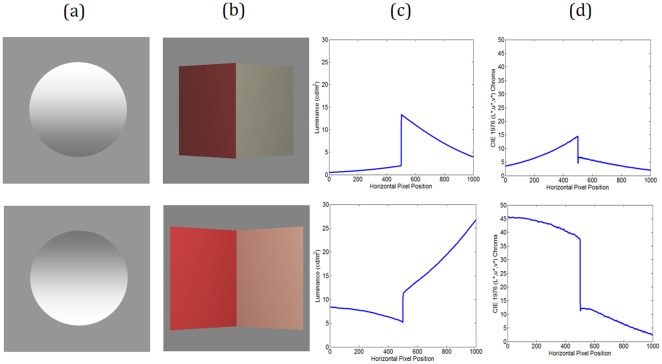
Examples of colour and luminance gradients associated with object shape. (a) Uniform luminance gradient makes the upper circle appear to be convex, and the lower one concave (or at least less convex). (b) The ‘colour Mach card’ depicting a convex roof at the top and a concave corner below (both card angles 50 deg). Notice the complexity of the colour and luminance gradients for these realistically rendered objects. (c) Horizontal luminance profiles for roof (top) and corner (bottom) and (d) chroma for roof (top) and corner (bottom) are very different (card angle 50 degrees).

Shading is a powerful cue to shape, especially in the presence of other depth or shape cues, such as perspective outline, texture or cast shadows [1], [2], [3], [4], [5], [6], [7]. For example, a vertical shading gradient across a circle delivers a compelling sense of depth (a convex sphere), even when the gradient is not necessarily physically accurate [8] (Figure 1a). Further, switching the direction of the gradient results in a switch to perception of a concave sphere (compare Figure 1a, upper, with Figure 1a, lower). This requires that the brain assumes light is coming from a single direction [2], [9]. The specificity of the perceptual effects observed has been linked to a tendency to perceive objects as if light comes from above [10], [11], [12], [13] (known as the ‘light from above prior’). The strength of this cue to shape, at least when in combination with other cues such as outline, is clear when one considers that many studies have used ‘painted’ shading gradients, like the simple linear shading gradient applied to the shapes in Figure 1a. Studies using simple gradients are important, but they do not capture the complexity of the light fields in real scenes [14]. The gradient of luminance and/or colour across an object is a complex combination of the object shape, the material it is made of and the light source form and location. Shading gradients alone, even in principle, correspond to multiple possible shapes [15], [16], [17], [18]. Because of this, shading is usually considered an ambiguous cue to shape unless it occurs in the presence of additional shape information [9]. Under such conditions, the visual system does treat it as an independent cue, that can be optimally combined with other shape cues [7].

Here we consider a realistically lit 3-D object, a card ‘folded’ at a colour border, portraying either a convex (roof shape, see Figure 1b top) or concave (corner shape, see Figure 1b bottom) dihedral angle. We used a physically accurate computer rendering technique to portray the full effects of light from a specific direction, including inter-reflections across the object itself [19]. A real world version of this ‘colour Mach card’ has previously been used to demonstrate that perceived object colour depends on perceived 3-D object shape, via the effect of mutual illumination [20], and subsequent studies have backed this up [21], [22]. Here, we explore a problem that is almost the inverse of this: whether the luminance (Figure 1c) and colour (Figure 1d) gradients on the realistically rendered card, that are very different for roofs and corners, can be interpreted appropriately by the visual system as a cue to the 3-D shape of the card. In particular, we explored the extent to which gradients are a useful cue when other cues are not available, but where observers are allowed to learn the relationship between shading gradient and shape, prior to experimental measurement. Previous work has suggested that the human visual system is sensitive to both the luminance and chromatic information contained in such gradients and that complex illumination features such as inter-reflections may be useful for unambiguous shape perception [23].

Unlike much other work [9], [11], [24], in this study we used realistically rendered, colour stimuli that deliver the full richness of real scenes. We chose a vertically oriented object, with smooth horizontal depth variation, and therefore largely horizontal gradients (Figure 1c), often delivering ambiguous shape perception [25]. We asked a number of novel research questions here:

First, is the visual system capable of utilising illumination gradients as a cue to shape without any other cues, and without prior exposure to the stimulus? Because the gradient cue is ambiguous, to do this the visual system would have to use ‘built-in’ prior knowledge to make use of the cue information. Second, if visual priors alone are *not* sufficient to make use of gradients, can the information required to disambiguate the gradient be easily learnt? For example, observers may need some exposure to the scene before the experiment to estimate properties such as the light position before they can use the gradients. To answer these two questions we tested perception of object shape, using gradient information only, for two groups of observers: one that had no prior experience of the stimuli, and one that was shown a short video containing a version of the stimulus with both gradient and outline information (similar to Figure 1b).

**Figure 2:**
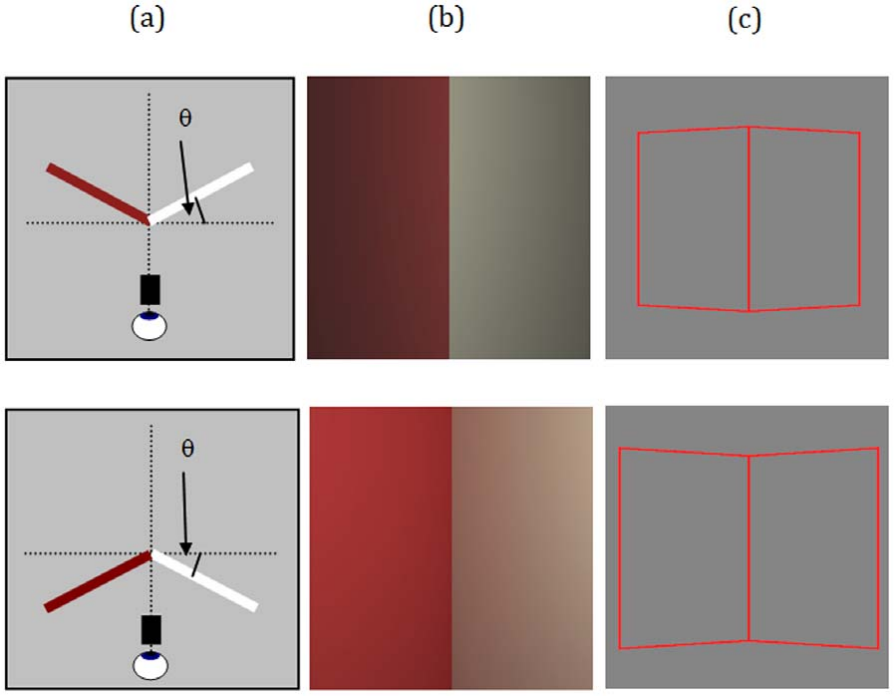
Stimuli and viewing arrangement. (a) Cartoon showing the colour Mach card, card angle θ, and viewing arrangement (not to scale) for roof (top row) and corner (bottom row) configurations. Note monocular viewing aperture. (b) Colour and luminance gradient stimuli for the cards used (top: roof, bottom: corner, angle 50 deg). (c) Outline cue stimuli, top: roof, bottom: corner, angle 50 deg.

Our third question explored learning of the shape-gradient mapping further. Here we asked whether observers were able to learn an arbitrary mapping between shape and gradient (essentially a test of memory), or whether there is a deeper connection between shape and gradient, resolving ambiguity by exploiting prior knowledge about gradients, that allows the visual system to use the gradient as a cue to shape. To investigate this, we tested groups of observers who were exposed to videos containing simpler, pared down, versions of the stimuli: one group saw a video where the average luminance and chromaticity of each side of the card was applied uniformly over the relevant side (preserving the effects of shape on the cards' colour/luminance, but removing gradients); a second group saw a video containing only a wire frame version of the stimulus, with no gradients or colour information, similar to Figure 2c. A third group were exposed to video containing stimuli with incongruent gradients, corresponding to the inverse stimulus shape. This last manipulation was designed to test if observers learned unrealistic mappings between gradient and outline, as well as mappings consistent with real lighting conditions.

Our fourth and fifth questions explored how well the visual system is able to exploit complex gradients. We considered if a similar magnitude of 3-D shape is perceived for the corner and roof, despite the very different physical patterns of gradient information available (Figures 1c and 1d). Finally, we investigated whether the shape perceived is consistent across cues, by comparing shape settings made using the gradient with those made using a different source of information, that from perspective outline. Although similar shape settings across cues are not guaranteed, even if the gradients are used as a genuine visual 3-D shape cue, similar shape settings would indicate that observers can do more than simply discriminate between the stimulus gradients.

## Results

‘Gradient-only’ stimuli were realistically rendered coloured Mach cards, presented on a CRT, viewed monocularly via a small tube, or viewing port (Figure 2a), and were composed of only the correct luminance and colour information, which we will call the ‘gradient cue’ (the object/screen boundaries were beyond the field of view, as in Figure 2b). Observers estimated the angle of a ‘corner’ or ‘roof’ card stimulus by adjusting the angle between two lines in a ‘view-from-above’ configuration, displayed on a separate monitor (see Methods). We used a wide range of card angles, θ (Figure 2a), from −70 deg (roof), through 0 deg (flat) to +70 deg (corner).

If observers are able to resolve the complexity of the gradient information and use it as a consistent shape cue, we expect them to be able to correctly and consistently set the shape to be either a corner or roof when presented with only the gradient cue, and without prior experience of our stimulus or lighting arrangement.

**Figure 3:**
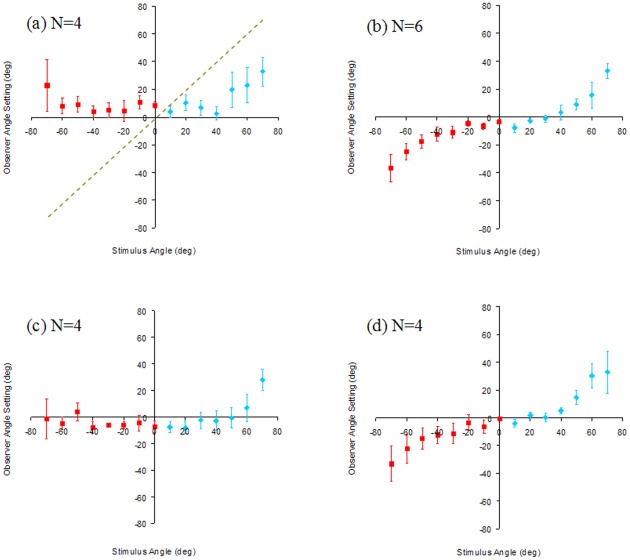
Observer shape settings (Gradient-only). Mean angle settings (averaged across observers) as a function of physical angle for roof (negative angle, red squares) and corner (positive angle, blue diamonds). Error bars show standard error of the mean. The dashed line on (a) indicates veridical performance for all figures. (a) ‘No Training’ (verbal instruction only). (b) ‘Full Training’ (short training video with a stimulus containing both gradient and outline cues, and correct setting lines). (c) ‘Outline Training’ (video with stimulus containing outline cue and setting lines only). (d) ‘Uniform Colour Training’ (video without detailed gradient cue, but correct mean colour and luminance on card surfaces, along with the outline cue and setting lines).

Figure 3a shows mean shape settings for four naïve observers that only received a verbal explanation of the task before starting the experiment, and had no knowledge of the specific gradients in the stimuli. We call this the ‘No Training’ group. Observer angle setting as a function of physical stimulus angle for roof (negative) and corner (positive) angles is plotted. There was some variation of setting with stimulus angle, with large angles being set as larger, but there were no negative average responses (ie setting consistent with the presence of the roof), despite half the stimuli specifying a roof. Observers were unable to reliably distinguish concave and convex shapes (Fishers exact test, per observer; SA: p = 0.8, AC: p = 0.5, CM: p = 0.2, WH: p = 0.2)

A separate group of six observers (‘Full Training’ group) performed the same experiment, but were shown a short video presentation prior to testing. This video showed a small, low resolution, version of the stimulus containing the gradient cue for the lighting conditions we used, but also bounded by a congruent outline cue (akin to figure 1b). Alongside the stimulus, the video also contained a top-down view demonstrating the correct setting line position that observers should set on the test-screen. Figure 3b shows the mean angle settings of the ‘Full Training’ group and delivers a very different pattern of response to that in 3a. Four of the six observers delivered settings that were clearly of opposite sign for roof and corner, and that increased with physical angle. These four observers were able to reliably assign roofs and corners to the correct category (Fisher's exact test; AC: p = 0.01, BM: p = 0.01, LM: p = 0.002, SC, p = 0.01), whilst the remaining two were not (LL: p = 0.3, MD: p = 0.54). For the four observers who could use the gradient cue, angle setting was a monotonic function of physical card angle, θ.

The majority of observers who learned via the ‘Full Training’ video were able to use the gradients to make consistent shape settings (Figure 3b), while none of those in the ‘No Training’ group were able to do so (Figure 3a).

The results so far suggest that observers in the ‘No Training’ group could not make consistent settings because they did not have sufficient visual information to disambiguate the gradient cue. Alternatively, these observers may have failed to understand the task correctly without viewing the training video. We tested this hypothesis by asking a further two groups of four observers to make settings after watching training videos containing either a wire frame version of the stimulus (‘Outline Training’) or a stimulus where the luminance and chromaticity of the card sides was averaged (for each stimulus angle independently) such that each side of the card was a uniform colour, containing a spatially coarse representation of the gradient information (‘Uniform Colour Training’). Figure 3c shows the mean settings of the ‘Outline Training’ group, who showed similar behaviour to that in Figure 3a. Three of the four observers in this group were unable to reliably distinguish concave and convex shapes (Fishers exact test; CH: p = 0.1, YR: p = 0.5, HH: p = 0.7). Results for one observer did show a significant ability to assign shapes to the correct category (Fishers exact test; NI: p = 0.05). Mean observer settings for the ‘Uniform Colour Training’ group are shown in Figure 3d. This training video provided enough information for the observers to make similar shape settings to the ‘Full Training’ group (Figure 3b), and again three of the four observers in this group were able to reliably assign roofs and corners to the correct category (Fisher's exact test; AC: p = 0.05, MA: p = 0.04, PC: p<0.001, CS: p = 0.1).

The settings made by the ‘Outline Training’ and ‘Uniform Colour Training’ groups suggest that observers must first learn some information about the scenes' characteristics and illumination in order to use the gradient cue, but exposure to detailed gradients are not needed to learn the required information.

**Figure 4:**
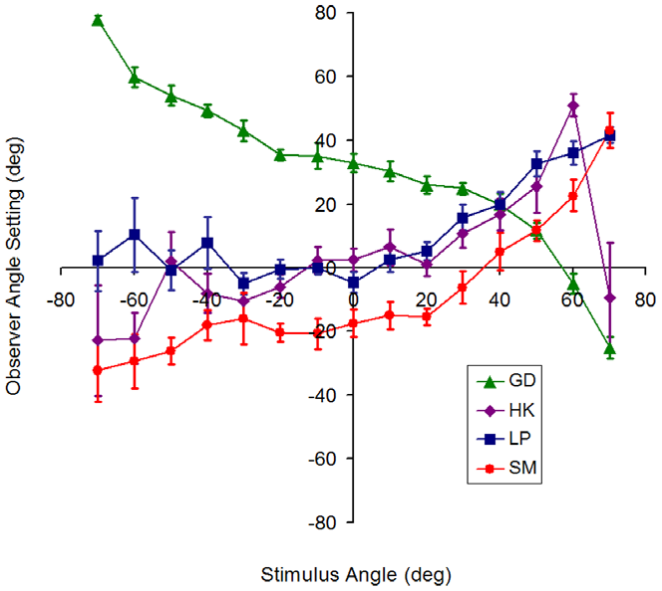
Observer shape settings (Gradient-only) for the ‘Incongruent Training’ group. Mean angle settings as a function of physical angle are shown for each of 4 observers separately. Error bars show standard error of the mean. ‘Incongruent Training’ consisted of a short video with the gradient cue indicating the opposite shape to the outline cue. For example: −40 degree shading displayed with +40 degree outline. Only one of the four observers (GD) made the ‘reversed’ pattern of settings that would be expected if they relied on memory of the training video.

We cannot yet conclude that observers can use the gradient information as a visual cue to shape: observers could be learning a simple mapping of gradient (or mean colour) to shape relying on their memory of the training video. To investigate if this was the case, we tested a group of observers who were trained using a video containing a version of the stimulus with incongruent shape cues, with the outline and gradient representing opposite shapes. For example a +40 degree (corner/concave) stimulus in the training video had a +40 stimulus outline and setting line position, but the shading from the −40 degree stimulus (roof/convex) was applied (‘Incongruent Training’). If observers make shape matches based on an outline-gradient mapping they learned during the training video, we would expect this group to make inverted settings, incorrectly assigning concave gradients to convex shapes and vice versa. In fact, inverted settings were made by only 1 of the 4 observers in the ‘Incongruent Training’ group. Figure 4 plots the results for this group, showing the mean settings for each of the four observers separately. The three observers who did not make inverted settings could reliably assign concave and convex shapes to the correct category (Fishers exact test; SM: p = 0.03, LP: p = 0.03, HK: p = 0.05) and performed similarly to those in the ‘Full Training’ group (Figure 3b). The fourth observer made inverted shape settings as if relying on memory of the training video and did not reliably assign the shape to the correct category (Fishers exact test; GD: p = 0.2). This result provides evidence that, for the majority of observers in the ‘Incongruent Training’ group (3/4), learning affords an opportunity to establish assumptions about the scene that allow them to correctly use the gradient in the stimuli as a shape cue, rather than making their settings based directly on the gradient-shape correspondence seen during training (i.e. using memory). It seems likely that assumptions about illuminant position and surface reflectance are established during the learning of the gradient information, in order to disambiguate the gradient cue.

**Figure 5:**
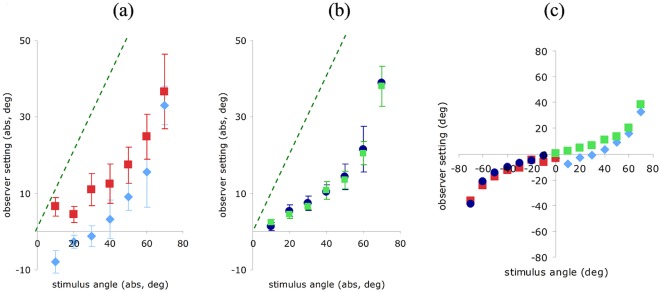
Comparisons of depth settings for roof and corner shapes, and gradient and outline cues. Average unsigned angle setting as a function of stimulus angle for (a) Gradient-only roof (red squares) and Gradient-only corner (blue diamonds); (b) Outline-only roof (green squares) and Outline-only corner (blue circles). Error bars show standard error of the mean. The dashed line indicates veridical performance. (c) Shows signed angle setting for corners and roofs, for both gradient and outline cues (same symbols as (a) and (b)).

We now consider in more detail the shape settings that were made by observers under ‘Full Training’. Despite there being very different luminance and chroma profiles across the concave and convex stimuli for each card angle (Figure 1c, 1d), observers were remarkably consistent in their settings when roofs and corners were compared for each angle. Figure 5a plots average unsigned angle setting from all observers, as a function of card angle, for gradient only stimuli. Notice that the roof (red squares) was consistently perceived as having a larger angle (steeper card with more depth) than the corner (blue diamonds). A two-way repeated measures ANOVA revealed that the difference between them was significant (F(1,5) = 7.007, p = 0.046). This difference is specific to the gradient cue. We verified this by testing perceived shape when observers' viewed stimuli containing only an object outline cue, consistent with the shape of the coloured Mach card (Figure 2c). The settings made by the ‘Full Training’ group in this ‘outline-cue-only’ test condition are shown in Figure 5b. Here there is very close overlap between observer settings for the roof (green squares) versus the corner (blue circles) and no significant difference between them (F(1,5) = 1.938, p = 0.223). Therefore, the differences observed in Figure 5a must be specifically linked to use of the gradient cue.

Our final question was to consider the extent to which the two cues delivered similar perceived angles, despite being very different cues to depth. The variance of settings made using the gradient was, for all stimulus angles, higher than that of the settings made using the outline (error bars in Figure 5a and 5b, show standard error of the mean. Mean variance across all observers and all card angles - gradient: 315 deg^2^, outline: 27 deg^2^). Thus the gradient appears to be a less reliable source of shape information than outline. However, mean shape settings are similar for both types of stimuli. Figure 5c compares roof settings for gradient only (red squares) and outline only (blue circles) cues, and corner settings for gradient and outline cues (blue diamonds and green squares, respectively). There is close overlap between the datasets, especially for roof settings. Although the shape settings are based on very different visual information, settings are similar, suggesting that the visual system is interpreting both sources of information as cues to almost the same shape. Note that observer settings of card angles are consistently underestimated for both corner and roof and also for both gradient and outline cues (Figures 3, 4 and 5). This is consistent with other literature: slant perception tends to be biased towards perceiving surfaces as flatter (more frontoparallel) than physically presented [1], [26], [27], [28], [29].

## Discussion

### Do ‘built-in’ priors provide enough information to allow the use of gradients as a cue to object shape, and if not, can the information be easily learnt?

Luminance and colour gradients are inherently ambiguous cues to shape, dependent on object shape, material, and the lighting environment. The literature on the use of shading as a depth cue is not conclusive on what prior information or assumptions are required by the visual system to effectively use these cues. While classical computer shape-from-shading algorithms (for example [15]) typically require knowledge of light position and surface reflectance properties in order to calculate local surface orientation, it is not clear if this is also the case for the human visual system. Some work has shown that the visual system may not use these assumptions and instead rely on a process dependent on global properties of shading on 3D shapes [4], [30], [31], [32]. However, it has also been shown that humans are able to judge, and are sensitive to changes in, light position in 3D scenes (for example [23], [33], [34]), and may estimate illuminant position [35], [36]. The results presented here suggested that observer can quickly and reliably learn about the relationship between themselves, the object reflectance and the light source, so as to disambiguate the gradient cue.

Observers in the ‘No Training’ group did not have an opportunity to learn anything about the stimulus before making shape settings using only the gradient cue. Results from this group (Figure 3a) showed that without prior exposure to the stimulus, observers are unable to make reliable shape settings, indicating that visual priors, or shape extraction mechanisms using only global shading and 3D shape properties are not sufficient to disambiguate the gradient cue. Observers in the ‘Full Training’ group had an opportunity to learn assumptions about illuminant position and surface reflectance before the experiment started. The short learning phase, with a time course of just 30 seconds, appeared to provide enough information to set the correct assumptions about reflectance and lighting, and to later interpret complex changes in gradient (Figure 1c, d) across the scene as a shape cue (Figure 3b). This was possible without the addition of any other shape cues (except during the learning phase). For the majority of participants who received full training, there was no ambiguity in responses over whether gradient depicted a convex roof, or a concave corner.

### What do observers learn and how do they make their settings?

The settings made by the ‘Full Training’ group showed that observers can make use of the gradient cue, given sufficient information prior to making their settings. However, this result alone cannot tell us if observers learn an arbitrary mapping between shape and gradient from the video training, or if they are really able to make and use suitable assumptions about lighting and object properties to understand the connection between shading gradients and shape. To answer this question, three further groups of observers made shape settings. Each of these groups was provided with different information about the gradients during the training video. In the ‘Outline Training’ group, observers were trained using a video describing the task and showing a representation of the stimulus without any shading (wireframe). This provided more visual information about the task than that received by the ‘No Training’ group, but also did not provide any information about the shading on the stimulus. Observers in the ‘Outline Training’ group, like those who received no training, performed poorly when making settings using the gradient cue alone (Figure 3c). This suggests that in the ‘Full Training’ case, the training video provides not only an aid to understanding the task, but also visual information about the scene that is needed to make accurate shape judgements.

The ‘Uniform Colour Training’ video contained a version of the stimulus with a spatially coarse representation of the shading, such that no gradients were present across the card (each side was of uniform colour and luminance), but the mean luminance and chromaticity of each side was as the same, for each card angle, as those in the ‘Full Training’ video. When trained using this video, observers performed as well as those exposed to the ‘Full Training’ video. This suggests that observers do not need to see the specific gradients in order to form the required assumptions about lighting and surface reflectance. The assumptions gained, based on the simplified video, are enough to allow them to use the gradient information available in the experimental stimuli to successfully establish shape.

The final group of observers were shown the ‘Incongruent Training’ video, which was specifically designed to test if the training could result in a learnt mapping between *any* gradients and shape. In this video, the stimulus contained gradients for the inverse of the shape given by the object outline (the setting lines in the video were appropriate for the outline cue, not the gradient cue). Observers who made ‘memory matches’ of gradient to shape would therefore be expected to make reversed shape settings. Three of the four observers did not make reversed settings, but instead performed similarly to those who were trained using congruent stimuli. This behaviour demonstrates that these observers were using the gradients in the stimuli as a visual cue to shape when making their settings.

### How well does the visual system exploit the gradient cue to shape?

We have shown that despite rather different forms of gradient and changes in gradient with shape, for convex roof and concave corner stimuli (see examples in Figure 1c and 1d) similar shapes were perceived for each (Figure 5). The gradients in the roof stimuli are due to intensity drop-off with distance from the light source, while the ones in the corner stimuli are also influenced by inter-reflections between surfaces and shading. Our visual system successfully assigns approximately correct shape in both situations, lending credence to sophisticated shape from shading algorithms that have shown that inter-reflections are in fact a possible source of useful 3-D information [37], [38] over traditional algorithms [15] where inter-reflections/mutual illuminations are ignored and lead to incorrect shape estimates.

We do see some asymmetries in Figure 5a and 5c, particularly for small angles, but this is more likely because of luminance ‘drop-off’ towards the edges of the stimuli due to our choice of light position, which was relatively close to the stimulus (see Methods). If the visual system were using the assumption that darker regions of an object were further away [39], [40], this effect would contribute to a negative offset in observer settings when card angles were small.

Although settings made using the gradient have a higher variance than those made using the outline, the perceived shape is remarkably consistent across the two cues for a very wide range of card angles (Figure 5c). Shape settings for the gradient cue were similar to those for a stimulus containing only perspective outline, indicating that observers are able to do more than simply discriminate between different gradients. Rather, with only a short learning period, observers can use gradients as a shape cue, almost as consistently as they use outline.

### What visual information forms each cue?

Use of perspective outline as a shape cue requires the assumption that the card being viewed is rectangular and that deviations from rectangular in the image are due solely to changes in 3-D shape. A body of evidence suggests that our visual system does this and that we assume that trapezoidal distortions are due to rotations in depth [41], [42], [43], [44], [45]. We are most familiar with this assumption when viewing the Ames Window (or trapezoid illusion: a frontoparallel trapezoid is interpreted as a rectangle in depth [46]), where the 3-D rectangle assumption causes the perception of non-rigid rotations. It is therefore perhaps not surprising that settings for the task with outline cue only, which requires a pair of abutting trapezoids to be interpreted as rectangles in depth (Figure 2c), are consistent and reliable.

Far more assumptions are required to interpret the gradient cue as arising from a pair of slanting surfaces. Gradients of colour and luminance across an image are a complex combination of the light source form, its direction, the object's 3-D shape and reflectance. The example gradients shown in Figure 1 will therefore be most likely for the specific stimulus and lighting parameters we specified in the rendering. Observers must make assumptions about the reflectance properties of the card surfaces and about the light position in order to use the gradient cue correctly. If these assumptions are very different from the true scene parameters, then perception will be inaccurate. Because our observers set equivalent angles, close to those made with the outline cue, in both the ‘Full Training’ and ‘Uniform Colour Training’ groups, we infer that the visual system must have learned a reasonable set of assumptions about the scene, with only limited training that does not need to have the same detail level as the stimuli. Additionally, because the majority of observers do not make inverted settings when trained using incongruent gradients (specifying the inverse shape), we believe that observers have learnt assumptions about the light environment and scene, rather than learning a simple mapping between outline and gradient.

### Discriminability of the two cue types

We considered whether each of the cues, gradient and outline, were set at a discriminable stimulus level for all of the angles studied. For the gradient cue this is difficult to establish, the literature is far from exhaustive and gradients can have very different profiles (Figure 1c and 1d) that could lead to different perceptual thresholds. In a previous paper [23] we characterised gradients by their total cone contrast [47] which allowed us to specify the strength of a colour change independent of observer and stimulus details [48]. In that study we showed that gradients of above 4% total cone contrast were discriminable. In the current study, the smallest gradient was present on the coloured side of the card for an angle of +20 degrees (concave) and resulted in a cone contrast of 5% while the minimum difference between total cone contrast for two consecutive angles (+20 and +30 degrees, coloured side of the card) was 12%. This suggests that all our current gradient stimuli should be discriminable from each other.

For the outline cue, there is more specific literature to consider when determining the discriminability of our stimuli. It has been shown that people can precisely judge the apparent slant of a frontoparallel trapezoid, and they behave as if it is a slanted rectangle [49]. These thresholds depend on size; for objects of similar size to our stimuli, detection thresholds are around 10 degrees [50].

One study has measured thresholds for obtaining slant angle of a single flat card, tilted vertically about a horizontal axis [51]. For both 2-D and 3-D task, thresholds were around 10% (expressed as Weber fractions), and biases were consistent with perceived depth being somewhat flattened. In our study, each stimulus was separated from its nearest-slant neighbour by at least 15%, making us confident that the stimuli were highly discriminable from one another. Error bars in Figure 5b show the variability of observer responses to be low, around the 10% expected.

### Biases in set angle

Almost all literature on slant or associated depth perception delivers estimates that are consistent with flattening of the perceived slant/depth compared to the physical stimulus [52]. As many have argued, the tendency of observers to underestimate the stimulus depth, evident in all our experimental conditions, could be attributed to residual cues to flatness, since stimuli were presented on a flat computer monitor [27], [28]. While we attempted to minimise the effects of other cues to depth by use of a viewing aperture, some cues to depth such as ocular accommodation cannot easily be removed and are set at values corresponding to a flat surface. Examination of our data for the ‘Full Training’ group shows that, in general, for both gradient and outline cues, observers rarely make settings indicating the reverse shape to the stimulus (e.g. concave settings for convex stimuli and vice versa), and very rarely overestimate the depth of the stimulus. The apparent bias towards flatter perceived shape cannot therefore be due to the averaging of settings based on ambiguous perception.

Our results concur with many studies that have explored monocular cues to the shape of dihedral angle stimuli using a range of different types of stimuli and depth cues and found apparent biases towards lower than veridical perceived depth or frontoparallel surfaces [4], [27], [28], [53] and general biases towards compression of depth with increasing distance [54]. In particular, it has been shown, using texture cues, that angles are misperceived such that objects look flatter than veridical [29]. Such a bias towards frontoparallel surface orientation could be explained in the Bayesian framework by the influence of prior knowledge about the distribution of likely surface orientations. In the case of our results, the larger bias for lower angles suggest that such a prior may be a heavy tailed distribution, but further investigation and modelling will be required to confirm this [55]. If a prior for frontoparallel surface orientation does indeed influence the setting made by our observers, the similarity of shape perception across cues (Figure 5c) is a potential surprising result. Since gradient seems to be a less reliable cue than outline (settings made using the gradient cue have higher variance, see results section), it might be expected that the influence of such a prior would be greater in the Gradient-only condition. However, this would only be the case if a single, common prior were associated with both cues.

### Conclusion

In this paper we studied the use of the complex luminance and colour gradients, present in the surface shading of realistic objects, as cues to three dimensional object shape. Despite very different shading for different object shapes, we showed that the visual system can use this information alone as a full depth cue, providing similar perception of object shape to an unrelated depth cue (outline perspective). However, this cannot be achieved using only visual priors to disambiguate gradient information, and some learning of the stimulus and scene properties is required. We explored learning in detail, and demonstrated that, even with a very short learning phase, and incomplete visual information, observers can quickly learn assumptions about the scene and lighting arrangements that allow gradients to then provide a powerful cue to shape. This work demonstrates that the visual system effectively solves the difficult problem of obtaining shape from surface orientation despite complex illumination in real scenes.

## Materials and Methods

### Stimuli

Stimuli were displayed using a Cambridge Research Systems ViSaGe system running in 42bit colour mode (http://www.crsltd.com/catalog/visage/overview.html), driving a calibrated Mitsubishi Diamond Pro 2070 CRT monitor. Two sets of stimuli were created, one for each test condition of the experiment:

1. Gradient-only stimuli: The ‘folded card’ stimuli consisted of a surface with a vertical central fold, one side white and the other coloured red, rendered at 1000×1000 pixels to cover the full screen with a 15.5 degree diameter field of view (Figure 2b) displayed at a scaled resolution of 632×949 (the horizontal resolution available is halved when using the CRS ViSaGe in 42bit colour mode). A range of card angles were used in the experiment (the angle of the card surfaces to the horizontal axis perpendicular to the viewing direction) ranging from −70° (‘roof’ shape) through 0° (‘flat’ shape) to +70° (‘corner’ shape), in steps of 10° (Figure 2a). The materials used for rendering the card surfaces were chosen from the Natural Color System (NCS) papers (http://www.ncscolour.com/). The use of real materials, with existing spectral surface reflectance data, allowed surface colours to be easily defined for physically accurate spectral rendering [19]. NCS_S0300N was used for the white card side and a red coloured paper (NCS_S0580Y90R) for the coloured card side. This particular red colour was chosen due to a high reflectance and high saturation, resulting in a large amount of chromatic mutual illumination on the white side of the Mach card. Surfaces were rendered under a D65 spectrum light source that consisted of a spherical point source positioned in front, above and slightly to the right of the stimulus (x,y,z of 13.33, 16.66 and −66.66 cm respectively) such that significant gradients due to mutual illumination and luminance fall off towards the edges was present in the stimuli, and varied with card angle.
2. Outline-only stimuli: Wire frame stimuli (Figure 1c), with veridical outlines for a card that had sides 10×10 cm in size, viewed from 1 m distance (the same as the actual observer viewing distance). For the flat card this corresponded to a rectangle of 11.4 by 5.7 degrees. The same range of angles as the Gradient-only condition were used. These stimuli were not rendered but drawn in real-time by the CRS ViSaGe system. The lines of the outline only stimuli were red (RGB [255,0,0]) and had a luminance of 23 cd/m^2^. Stimuli were displayed on a grey background with a luminance of 10 cd/m^2^.

All stimuli were viewed via a viewing box, 1 m in length, height 48 cm and width 50 m (to fit exactly around the CRT). Observers looked through an aperture (tube 3 cm diameter, 10 cm length, 15.5 degree diameter field of view) to ensure monocular viewing and the exclusion of unwanted depth cues.

### Observers

22 naive observers (mean age 38) took part in the experiment, 17 females, 5 males. All subjects had normal colour vision (verified using the Farnsworth-Munsell 100 hue test) and normal or corrected to normal acuity. Observers all gave informed written consent before taking part in the study. Experimental procedures followed the ethical guidelines issued by the Bradford School of Optometry and Vision Science and approved by the Ethics Panel of the University of Bradford.

### Apparatus and Procedure

The experiments were controlled by Matlab software, using the CRS Toolbox (http://www.crsltd.com/catalog/vsgtoolbox/index.html) and the Psychophysics Toolbox [56]. Observers were asked to adjust a top-down view of the card, on a separate computer monitor, so that the angle separating one side of the card from the other was the same as that perceived in the viewed stimulus (similar to the method used by other authors [29]). In the top-down view, lines were 2 mm wide by 10 cm long (the full ‘card’ was 20 cm wide when flat). Line and background colour and luminance were matched to that of the Outline-only stimuli. The top-down view was also viewed from 1 m. Observers were shown a brief video demonstration of how the task should be performed before beginning the first session. This consisted of a short video presentation containing images of the stimulus and also the setting task lines. The video showed the stimulus, and the corresponding view-from-above setting line configuration, vary slowly throughout the full range of stimuli present in the experiment (two cycles over 30 seconds showing angles from the steepest convex to the steepest concave angle and vice versa). The ‘view from above’ task was explained verbally at the same time, using the video demonstration for reference. Four separate training videos were used with different groups of observers. In each video the representation of the stimulus contained different information: ‘Full Training’ - both outline and gradient cues; ‘Incongruent Training’ - outline cue plus a conflicting gradient cue from the reverse shape (i.e. +40 degree outline was paired with −40 degree shading); ‘Uniform Colour Training’- outline cue plus uniform coloured stimulus surfaces that match the mean luminance and colour of the gradient cue; ‘Outline Training’ - outline cue only. A fifth group of observers did not see a training video before the experiment (‘No Training’).

Observers performed the experiment sequentially for each experimental condition (order was random across observers). Each condition required 150 observer estimates, ten for each of the available card angles. Observers were allowed as much time as they wished to make each setting, but were encouraged to perform the task as quickly as possible, making estimates from their initial impressions of object shape. Typically each session was completed in around 25 minutes.
